# Long-term pathological and immunohistochemical features in the liver after intraoperative whole-liver irradiation in rats

**DOI:** 10.1093/jrr/rru005

**Published:** 2014-02-23

**Authors:** Masumi Imaeda, Hitoshi Ishikawa, Yukari Yoshida, Takeo Takahashi, Yu Ohkubo, Atsushi Musha, Mayumi Komachi, Yoichi Nakazato, Takashi Nakano

**Affiliations:** 1 Department of Radiation Oncology, Gunma University, Graduate School of Medicine, 3-39-22 Showa, Maebashi, Gunma 371-8511, Japan; 2 Department of Radiation Oncology, University of Tsukuba, Faculty of Medicine, 1-1-1 Tennodai, Tsukuba, Ibaraki 305-8575, Japan; 3 Department of Radiation Oncology, Saitama Medical University, 1981 Kamodatsujido, Kawagoe, Saitama 350-8550, Japan; 4 Department of Pathology, Hidaka Hospital, 886 Nakao, Takasaki, Gunma 370-0001, Japan

**Keywords:** radiation-induced liver injury, liver fibrosis, transforming growth factor-beta 1, alpha smooth muscle actin, apoptosis

## Abstract

Radiation therapy (RT) has become particularly important recently for treatment of liver tumors, but there are few experimental investigations pertaining to radiation-induced liver injuries over long-term follow-up periods. Thus, the present study examined pathological liver features over a 10-month period using an intraoperative whole-liver irradiation model. Liver function tests were performed in blood samples, whereas cell death, cell proliferation, and fibrotic changes were evaluated pathologically in liver tissues, which were collected from irradiated rats 24 h, 1, 2, 4 and 40 weeks following administration of single irradiation doses of 0 (control), 15 or 30 Gy. The impaired liver function, increased hepatocyte number, and decreased apoptotic cell proportion observed in the 15 Gy group, but not the 30 Gy group, returned to control group levels after 40 weeks; however, the Ki-67 indexes in the 15 Gy group were still higher than those in the control group after 40 weeks. Azan staining showed a fibrotic pattern in the irradiated liver in the 30 Gy group only, but the expression levels of alpha smooth muscle actin (α-SMA) and transforming growth factor-beta 1 (TGF-β1) in both the 15 and 30 Gy groups were significantly higher than those in the control group (*P* < 0.05). There were differences in the pathological features of the irradiated livers between the 15 Gy and 30 Gy groups, but TGF-β1 and α-SMA expression patterns supported the gradual progression of radiation-induced liver fibrosis in both groups. These findings will be useful in the future development of protective drugs for radiation-induced liver injury.

## INTRODUCTION

Surgery represents a known curative treatment method for patients with hepatocellular carcinoma (HCC) [1, 2], but it is often difficult to perform surgical resection for those patients displaying tumor invasion into the portal vein, those with multiple tumor lesions, or those with liver dysfunction caused by liver cirrhosis. In such cases, percutaneous ethanol injections or ablation therapies are performed for small tumors and transarterial chemoembolization typically for large tumors; regardless, curability of the cancer is unsatisfactory [2–4].

There is ambivalence about the role of radiation therapy (RT) in HCC because liver RT can induce severe liver dysfunction in patients [5, 6]. Recently, there have been reports regarding the efficacy of modern RT procedures, such as stereotactic body radiation therapy (SBRT) and charged particle therapy in liver tumors (including metastases derived from other primary sites) [7–10]. These highly conformal RT methods can three-dimensionally deliver a large dose to the tumor while sparing the surrounding normal liver tissue, thereby providing a method of controlling liver tumors with minimal liver dysfunction. Some investigators have also analyzed predictive factors, using dose–volume histograms (DVHs), for RT-induced liver dysfunction [10–12].

Recent advances in cellular and molecular biology have provided new insights into radiation pathogenesis mechanisms in a variety of late-responding normal tissues. Specifically, several experimental studies on lung injury induced by radiation exposure suggest that the extracellular matrix and transforming growth factor-beta 1 (TGF-β1) play key roles in the development of chronic fibrosis [13–15]. Although it is believed that TGF-β1 is the most important cytokine, also responsible for liver fibrosis [16, 17], the investigation of radiation-induced hepatic fibrosis has been limited because it is difficult to accurately and safely deliver large radiation doses to the liver by X-ray-delivered external beam radiotherapy. In particular, acute and late severe radiation-induced morbidities of the gastrointestinal tract prevented us from observing in detail the effects on long-term liver damage.

Here, we established a rat model to analyze liver damage over a long-term 10-month period following administration of large irradiation doses using an intraoperative technique. In addition, immunohistochemical staining was used in irradiated rat livers to examine various pathological changes, including hepatocyte cell death, proliferation, and expression of markers indicative of hepatic fibrosis, such as TGF-β1 and alpha smooth muscle actin (α-SMA).

## MATERIALS AND METHODS

### Liver irradiation

**Fig. 1.**
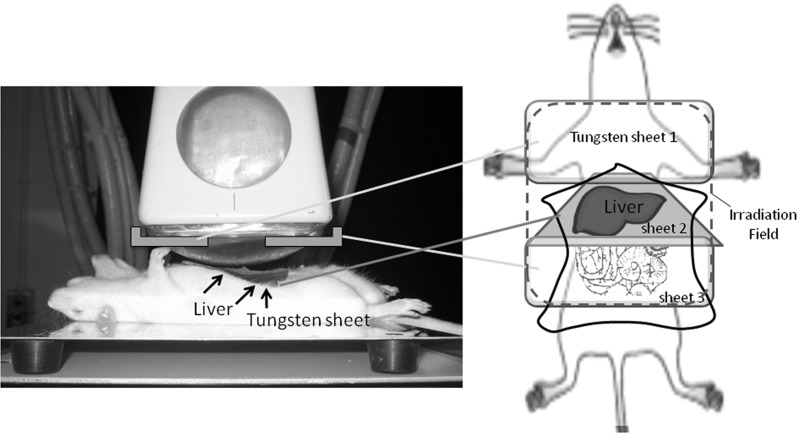
Schema for the whole-liver irradiation technique used in this study.

Six-week-old male Wister rats ∼180–190 g in weight were purchased from Charles River Laboratories (Japan). Before irradiation, each animal was anesthetized by intraperitoneal injections of pentobarbital (20 mg/kg) and the rat abdomens were opened surgically. A 3-mm-thick high-density tungsten sheet (density: 12, Nippon Tungsten Co. Ltd, Japan) was placed behind the liver to shield the gastrointestinal tract and lungs, and the livers were irradiated in supine-positioned rats, as demonstrated in Fig. 1. Single doses of 0 (control), 15 or 30 Gy were delivered with the Stabilipan 2 Orthovoltage Unit (Siemens, Germany) operating at 200 kVp with a 1 mm copper filter. At a distance of 30 cm, the dose rate was 1.47 Gy/min. The rats were warmed with a light heater to prevent hypothermia. After irradiation, the abdomens were surgically closed and the rats were carefully maintained with three rats per cage. The blood samples were obtained from the caudal vein and heart for the measure of albumin (ALB), aspartate aminotransferase (AST), and alanine aminotransferase (ALT) levels, as markers of liver damage; all blood tests were performed at the Mitsubishi Chemical Medience Corporation (Tokyo, Japan). The rats were sacrificed 24 h, 1, 2, 4 or 40 weeks post irradiation for investigation of liver pathology. All experiments were carried out according to the Animal Care and Experimentation Committee of Gunma University, Showa Campus (Maebashi, Japan).

### Histopathological and immunohistochemical analyses

The rat livers were fixed in 10% neutral buffered formalin, embedded in paraffin and sectioned (3 µm thickness). The mounted sections were subjected to hematoxylin and eosin (H&E) or Mallory–Azan staining. The paraffin-embedded tissue sections were deparaffinized and hydrated using xylene and graded ethanol. Sections were then placed in antigen retrieval solution (Target Retrieval Solution S1699, DAKO, Denmark), heated at 95°C for 30 min in a microwave oven, and cooled to room temperature for 10 min. After washing the sections, a 0.3% hydrogen peroxide solution was applied to block endogenous peroxide for 30 min at room temperature. The sections were then incubated in 10% goat serum in phosphate-buffered saline (PBS) for 30 min at room temperature to reduce non-specific antibody binding. Primary anti-TGF-β1 polyclonal (1:200, Santa Cruz, CA, USA), anti-α-SMA monoclonal (1:500, Abcam, Cambridge, UK), and anti-Ki-67 monoclonal antibodies (1:50, DAKO) were applied overnight in a humidified chamber at 4°C. Slices were washed in PBS and then incubated for 60 min at room temperature with the ready-to-use peroxidase-labeled secondary reagent, ENVISION + (DAKO). After rinsing with PBS, peroxidase activity was visualized using diaminobenzidine (DAB, DAKO). The slides were counterstained with Mayer's hematoxylin.

The TdT-mediated dUTP-biotin nick end-labeling (TUNEL) assay was performed using the ApopTag Fluorescein *In Situ* Apoptosis Detection Kit (Chemicon International, Temecula, CA, USA) according to the manufacturer's instructions. The DNA fragments were visualized using DAB, and the slides were counterstained with Methyl Green Solution. Five to eight fields per liver specimen were examined at random, and more than 1000 cells per specimen were counted to calculate the proportion of positive cells. Positive cell proportions in the irradiated liver samples were compared with those in the non-irradiated liver samples at the respective time-points.

### Statistical analysis

The data were presented as the means ± standard deviations (SDs). Comparative analysis of the three optimization steps was performed using one-way analysis of variance (ANOVA). *Post hoc* analysis was performed using the Student's t-test. Survival curves were generated by the Kaplan–Meier estimate [18] and were compared using the log-rank test. A *P*-value < 0.05 was considered statistically significant.

## RESULTS

### Survival of irradiated rats

**Fig. 2.**
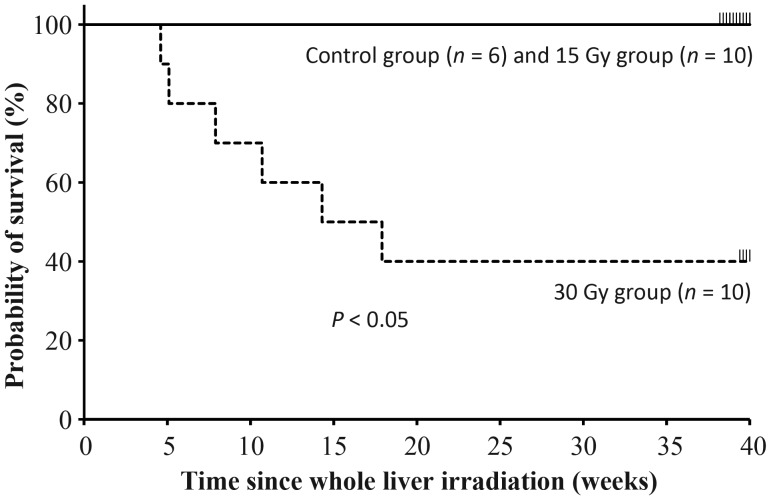
The survival curves of rats following whole-liver irradiation. No deaths in the control or 15 Gy groups were observed with irradiation, but 60% of the rats in the 30 Gy group died by 40 weeks post irradiation.

Whole-liver irradiation using single-fraction doses of either 15 Gy or 30 Gy was intraoperatively performed in 10 rats per group, and sham irradiation was performed in an additional six rats as the control group. The body weights and activity levels of the rats in both the 15 Gy and 30 Gy irradiation groups gradually decreased. Compared with the control group, there were significant weight reductions in the rats of both irradiated groups 8 weeks post irradiation (366.4 ± 16.3 g, 309.8 ± 51.4 g and 243.5 ± 71.9 g in the control, 15 Gy and 30 Gy groups, respectively, *P* < 0.01). Although the mean weight of the 30 Gy group was lower than that of the control group 40 weeks post irradiation, the mean weight of the 15 Gy group returned to the level of the control group (496.5 ± 19.3 g, 489.6 ± 34.5 g and 415.3 ± 14.0 g in the control, 15 Gy and 30 Gy groups, respectively). None of the rats in the control or 15 Gy groups died during the 40-week time course, but 60% of the rats in the 30 Gy group did (Fig. 2). Evidence of jaundice at autopsy was indicated by a yellow coloration in the skin and urine in 4 (67%) of 6 dead rats. Liver swelling was observed in all but one rat that died within the 8 weeks post irradiation, but no irradiation-induced changes in the gastrointestinal tract were observed, despite detection of ascites in some cases. On the other hand, deformed and shrunken livers and pleural effusions were observed in the rats that died later.

### Blood tests

Blood samples serially collected from six rats in each group were examined for liver function parameters. In the 30 Gy group, three of the rats died within 8 weeks after irradiation administration, and therefore, the 4- and 40-week post-irradiation data in this group were derived from the remaining four and three surviving rats, respectively.

Table 1 summarizes the ALB, AST and ALT plasma levels in each group of rats. The plasma levels of AST and ALT in the 30 Gy group were higher than those in the control group 4 and 40 weeks after whole-liver irradiation, and the AST level in the 30 Gy group after 40 weeks was significantly higher than that in the control group (*P* < 0.05). In addition, the decreased ALB levels seen in the 15 Gy group at the 2 week time-point returned to control group levels, whereas the ALB levels in the 30 Gy group were still significantly lower than those in the control group 40 weeks post irradiation.

**Table 1.** The results of blood test for liver function

Group	24 h	1 week	2 weeks	4 weeks	40 weeks
ALB					
Control	4.6 ± 0.2	4.2 ± 0.3	4.3 ± 0.1	4.2 ± 0.3	4.5 ± 0.1
15 Gy	3.4 ± 0.2*	3.3 ± 0.6*	3.3 ± 0.3*	3.8 ± 0.3	4.4 ± 0.2
30 Gy	3.2 ± 0.1*	2.5 ± 0.2*	2.4 ± 0.3*	2.8 ± 0.4*	3.7 ± 0.4*
AST					
Control	167.8 ± 70.7	136.1 ± 50.5	131.0 ± 34.5	161.5 ± 99.9	174.5 ± 89.9
15 Gy	131.0 ± 26.8	123.5 ± 79.2	139.3 ± 60.9	121.2 ± 55.0	344.8 ± 244.0
30 Gy	104.7 ± 24.1	164.3 ± 185.5	113.0 ± 55.1	269.0 ± 188.1	345.7 ± 127.3**
ALT					
Control	40.7 ± 8.3	37.7 ± 5.9	40.3 ± 3.9	54.0 ± 30.1	52.3 ± 30.1
15 Gy	32.3 ± 6.2	28.3 ± 6.9*	38.5 ± 3.4	52.2 ± 5.8	66.0 ± 7.7
30 Gy	32.0 ± 6.0	24.8 ± 2.9*	29.1 ± 9.2*	66.5 ± 47.4	205.0 ± 82.1

ALB = albumin, AST = aspartate aminotransferase, ALT = alanine aminotransferase. * indicates significantly lower than the control group (*P* < 0.05). ** indicates significantly higher than the control group (*P* < 0.05).

### Apoptosis and proliferation of hepatocytes

**Fig. 3.**
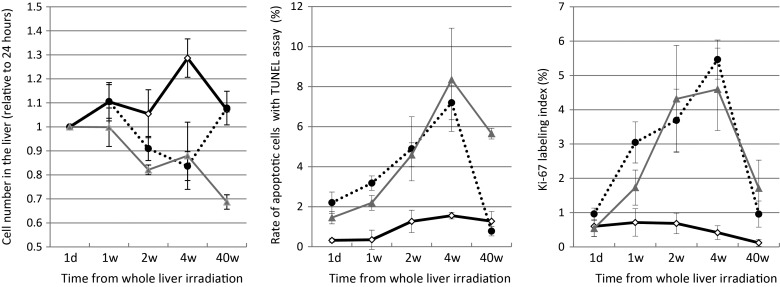
Pathological changes in liver cells following whole-liver irradiation for the respective time-points (control: bold line, 15 Gy: dotted line, 30 Gy: gray line). (**A**) The liver cell counts (relative to 24-h time-point) in the 15 Gy group after 4 and 40 weeks were 0.84 ± 0.15 and 1.08 ± 0.14, respectively, and the corresponding cell counts in the 30 Gy group were 0.88 ± 0.29 and 0.69 ± 0.07, respectively. The counts in the 15 Gy group 40 weeks post irradiation returned to the control group levels (1.07 ± 0.02). (**B**) The ratio of apoptotic cells in the irradiated groups significantly increased 4 weeks post irradiation (7.2 ± 0.9% in the 15 Gy group and 8.3 ± 2.6% in the 30 Gy group). The ratio in the 15 Gy group after 40 weeks (2.3 ± 0.6%) returned to the control group level (1.8 ± 0.9%), but that in the 30 Gy group remained at the high level (5.7 ± 0.4%). (**C**) The labeling indexes for Ki-67 immunohistochemical staining in the irradiated groups increased by 1 week post irradiation and reached the peak level after 4 weeks, irrespective of irradiation doses.

The number of hepatocytes was counted in five different central vein areas within the liver specimens from each rat at × 100 magnification per section, and the changes in the number of cells as a function of time since irradiation are shown in Fig. 3A. The cell counts in the irradiated livers were significantly decreased 4 weeks post irradiation, but after 40 weeks, the 15 Gy group cell counts returned to control group levels.

**Fig. 4.**
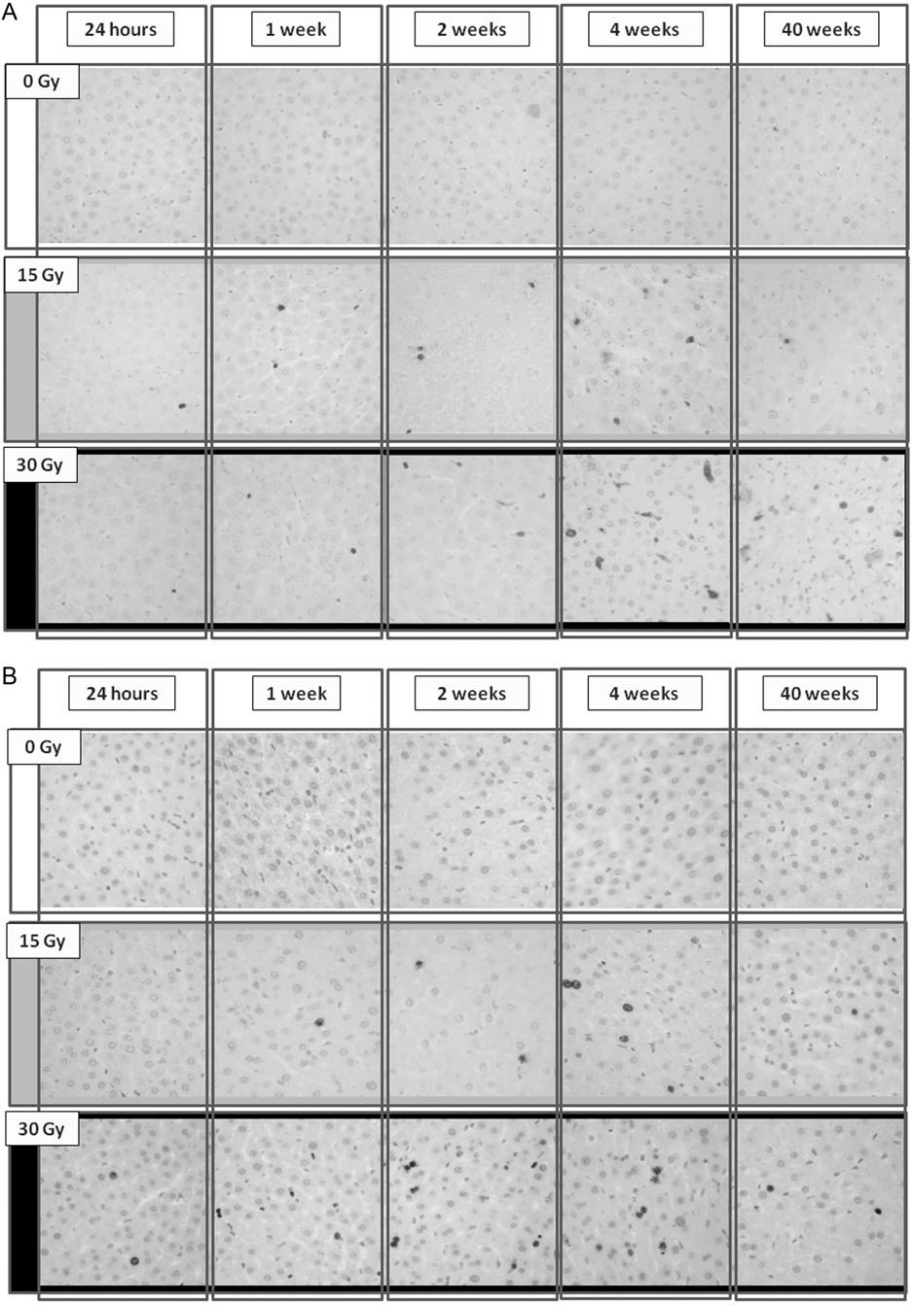
Changes in the (**A**) apoptotic ratio and (**B**) Ki-67 labeling index after whole-liver irradiation in the three rat treatment groups.

The number of hepatocytes undergoing apoptosis, caused by direct damage from intraoperative liver irradiation, were determined using the TUNEL assay in three rats from each group (Fig. 4A). The percentage of apoptotic cells increased in the 15 Gy and 30 Gy groups within the 4 weeks post liver irradiation in comparison with the control group (Fig. 3B). Notably, apoptosis was still evident in 5.7 ± 0.4% of the hepatocytes in the 30 Gy group 40 weeks post irradiation. On the other hand, the percentage of apoptotic cells in the 15 Gy group after 40 weeks decreased and returned to the level observed in the control group. Changes in the fraction of Ki-67-positive hepatocytes in the three treatment groups are summarized in Fig. 3C. The Ki-67 index in the irradiated groups was increased at all of the time-points tested (Fig. 4B).

### Liver fibrosis and protein expression of α-SMA and TGF-β1

**Fig. 5.**
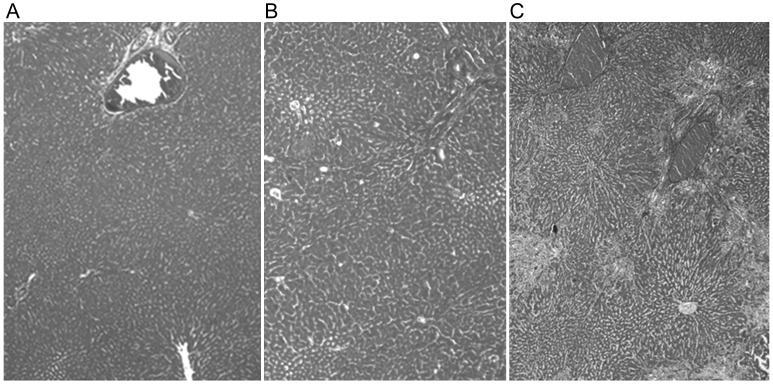
Azan staining 40 weeks post whole-liver irradiation in the (**A**) control, (**B**) 15 Gy, and (**C**) 30 Gy groups. Fibrotic changes were observed in both irradiated groups, but a high grade of bridging fibrosis was only observed in the 30 Gy group.

Microscopy and H&E staining showed that there were decreases in hepatocyte number, inflammatory cell infiltration, and necrotic foci number in the irradiated groups 40 weeks after liver irradiation. Azan staining showed a distortion in liver cellular architecture and a high grade of bridging fibrosis, but only in the 30 Gy group (Fig. 5).

**Fig. 6.**
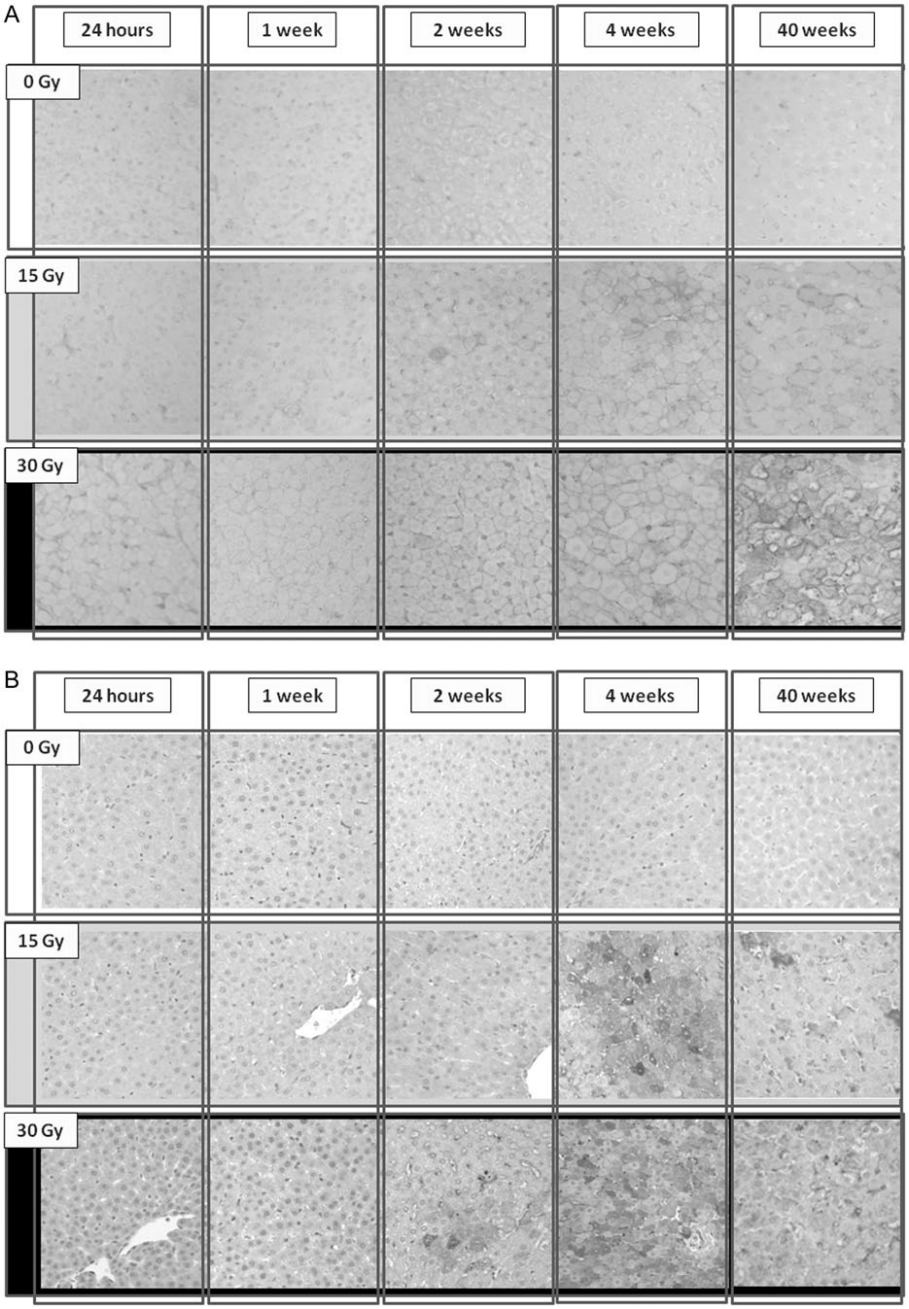
Changes in the expression of (**A**) TGF-β1 and (**B**) α-SMA after whole-liver irradiation in the three rat treatment groups.

The protein expression of α-SMA and TGF-β1 was investigated by immunohistochemical staining (Fig. 6A and B), and Table 2 indicates the expression levels of the three rat groups 24 h, 1, 2, 4 and 40 weeks post liver irradiation. The expression of α-SMA and TGF-β1 was significantly (*P* < 0.05) higher in the irradiated groups 2, 4 and 40 weeks after irradiation compared with the control group, and the expression of both markers 40 weeks post irradiation was significantly higher in the 30 Gy group compared with the 15 Gy group.

**Table 2.** Expressions of α-SMA and TGF-β1of irradiated hepatocytes

Group	24 h	1 week	2 weeks	4 weeks	40 weeks
α-SMA (%)					
Control	0.11 ± 0.06	0.25 ± 0.05	0.34 ± 0.24	1.08 ± 0.74	0.08 ± 0.07
15 Gy	0.37 ± 0.39	0.65 ± 0.18*	2.30 ± 0.36*	4.86 ± 0.76*	4.11 ± 0.68*
30 Gy	0.32 ± 0.17	0.57 ± 0.35	1.43 ± 0.51*	3.76 ± 2.19*	7.16 ± 4.30*
TGF-β1 (%)					
Control	0.3 ± 0.2	0.5 ± 0.1	0.5 ± 0.4	1.5 ± 0.3	8.0 ± 2.8
15 Gy	0.6 ± 0.9	5.0 ± 0.2*	8.0 ± 0.5*	29.1 ± 2.6*	18.6 ± 9.0*
30 Gy	2.3 ± 1.4	5.0 ± 0.9*	14.3 ± 2.7*	34.6 ± 12.1*	28.2 ± 5.4*

*P < 0.05.

## DISCUSSION

Irradiation of normal tissues results in fibrosis, which is believed to be the most universal radiation-induced late effect. Consequently, many studies have been designed using rat models to elucidate the histopathological changes that occur following liver irradiation [16, 17, 19–22]. Liver irradiation generally results in a loss of parenchymal hepatocytes and distortion of lobular architecture accompanied by pericentral and periportal fibrosis. However, most studies were performed over relatively short follow-up periods and with rat livers entirely or partially exposed to an external beam irradiated though the skin, potentially inducing gastrointestinal and liver injuries. Therefore, one of the aims of the present study was to establish a rat model using an intraoperative whole-liver irradiation technique that spares the surrounding normal gastrointestinal tract and skin tissues, in order to examine the irradiation-induced histological changes over longer periods. Our technique is evidently feasible, since all rat individuals in the 15 Gy group survived > 10 months following irradiation. In fact, the tungsten sheet used in this study was confirmed to shield > 98% of the irradiation; thus, the gastrointestinal irradiation dose was < 0.6 Gy, even in the 30 Gy group.

According to our results, apoptotic cells were significantly more abundant 1–4 weeks post whole-liver irradiation, and it could be speculated that the increase in cell death led to the decrease in the number of hepatocytes seen in both irradiated groups 2–4 weeks following irradiation (Fig. 3). However, the hepatocyte counts in the 15 Gy group returned to the baseline level after 40 weeks, whereas those in the surviving rats of the 30 Gy group continued to decrease after 40 weeks. The apparent differences between the 15 Gy and 30 Gy groups in hepatocyte number after 40 weeks could be explained by the differences observed in the apoptotic cell counts and Ki-67 labeling indexes between the groups; the elevated Ki-67 labeling index in the 30 Gy group after 40 weeks indicated continued proliferation of the hepatocytes. The liver irradiation-induced damages were still apparent in the 30 Gy group, whereas rats in the 15 Gy group had mostly recovered from liver damage by this time. Therefore, the considerably poorer survival curve in the 30 Gy group compared with the 15 Gy group was likely attributed to an imbalance between cell death and recovery. These results are supported by Geraci *et al*., who demonstrated a close relationship between irradiation dose and liver cell viability [23]. In their study, 3 months after administering 0–15 Gy irradiation doses, the parenchymal cell viability ranged from 80–90%, but a dose-dependent decline was evident, since only 39% of the cells were considered viable after a 25-Gy dose.

TGF-β is recognized as a key cytokine in the regulation of inflammation [24, 25], and overproduction of TGF-β1 is a major cause of radiation-induced tissue fibrosis in the liver as well as in the lungs [14–17]. Overall, the proportions of TGF-β1 positive cells in the irradiated groups gradually increased, peaking 4 weeks after irradiation and predominantly appearing in the 30 Gy group. Seong *et al*. reported a similar pattern in TGF-β1 levels after partial liver irradiation; the mRNA levels gradually increased to a maximum of 3.6-fold by Day 28, the latest time-point tested [17]. The present study examined the expression of TGF-β1 by immunohistochemical staining over longer periods and, interestingly, the upregulation of TGF-β1 was maintained even after 40 weeks of irradiation in a dose-dependent manner. Furthermore, our results for α-SMA, which is also a marker of liver fibrosis [26, 27], suggested a time-dependent effect of radiation on liver fibrosis induction; the further elevation in α-SMA expression in the 30 Gy group remained after 40 weeks and may indicate continual progression of liver fibrosis, ultimately resulting in death. On the other hand, the expression levels of TGF-β1 and α-SMA remained elevated even in the 15 Gy group 40 weeks after irradiation. Hence, the results may indicate that radiation-induced liver fibrosis in the 15 Gy group gradually progresses over 40 weeks, despite decreases in apoptotic cell number and the Ki-67 labeling index in the irradiated liver.

As mentioned above, we have challenged the establishment of a rat model using an intraoperative whole liver technique for long-term observation of radiation-induced liver injuries, but we cannot conclude whether this irradiation technique was the best method or not. Recent advances in external beam irradiation techniques for small animal research have stimulated the development of precise irradiation using micro-CT scans [28]. However, it seems difficult to deliver large irradiation doses to the whole liver without occurrence of severe surrounding normal tissue toxicities (even if the modern external beam irradiation technique is adopted) because of low spatial resolution of micro-CT and the treatment planning system and various errors including organ motion errors during the treatment [29]. On the other hand, intraoperative irradiation takes much time and effort and is more invasive than external beam irradiation, and it might cause acute damages to the liver and other organs, even in the control group. However, our technique seems to be feasible because none of rats died and there were no obvious changes detected in the blood test for liver function or the histopathological examination during the 40-week time-course in the group.

There were some limitations to the present study, e.g. 60% of the rats in the 30 Gy group died, and it could not be confirmed whether liver dysfunction was the cause of all of these deaths. As mentioned above, the gastrointestinal tracts were almost fully shielded from radiation, and while evidence of jaundice was seen in the rats, perforation of the tracts was not observed at autopsy. Furthermore, the tungsten shield factor in this study was > 98%. To validate the effect of the tungsten shield, we examined the apoptotic index of the small intestine crypts behind the shield using the TUNEL assay in three rats after irradiation, and the percentages of apoptotic cells in the control and 30 Gy groups after 4 h were 0.9 ± 0.1% and 1.5 ± 0.7%, respectively (*P* = 0.27). In addition, Chi *et al*. reported that the survival rates in rats 12 weeks after whole-liver irradiation with 20-, 40- and 65-Gy doses, administered by another intraoperative technique without the shield, were 100%, 50% and 25%, respectively, and most of the deaths occurred 5–25 weeks after irradiation [20]. Taking the results of that study into consideration, it is likely that most of the rats died of liver dysfunction in the present study. Another limitation was that the three rats from the 30 Gy group died within the 10 weeks post irradiation; therefore, for the ALB, AST and ALT measurements, the data from the 4- and 40- week time-points in this group were obtained from the remaining four and three surviving rats, respectively. There were no significant differences after 4 weeks in the AST and ALT levels between the 15 Gy and 30 Gy groups, although they were higher in the latter relative to the former. The results in the present study might have been skewed by the small subject number in the 30 Gy group. To validate our results, reinvestigation using an additional six rats was done over a 40-week follow-up, and three of these rats survived the full 40 weeks, which was in accordance with the first set of results.

## CONCLUSION

In conclusion, we have established in rats a radiation-induced liver damage model for long-term examination, and our results demonstrated pathological mechanisms for liver injury caused by irradiation. There were differences in cell number, apoptotic cell proportion, and the Ki-67 labeling index between the 15 Gy and 30 Gy groups after liver irradiation; the TGF-β1 and α-SMA expression results might support the gradual progression of radiation-induced liver fibrosis over the 40-week period following irradiation, even in the 15 Gy group. This model will be applicable for future work on the development of protective drugs for liver fibrosis following irradiation.

## FUNDING

This work was supported by Grants-in-Aid for scientific research from the Ministry of Education, Culture, Sports, Science and Technology (24591832) of Japan.

## References

[1] Takayama T, Sekine T, Makuuchi M (2000). Adoptive immunotherapy to lower postsurgical recurrence rates of hepatocellular carcinoma: a randomised trial. Lancet.

[2] Arii S, Yamaoka S, Futagawa S (2000). Results of surgical and nonsurgical treatment for small-sized hepatocellular carcinomas: a retrospective and nationwide survey in Japan. Hepatology.

[3] Lencioni R, Pinto F, Armillotta N (1997). Long-term results of percutaneous ethanol injection therapy for hepatocellular carcinoma in cirrhosis: a European experience. Eur Radiol.

[4] Rossi S, Di Stasi M, Buscarini E (1996). Percutaneous RF interstitial thermal ablation in the treatment of hepatic cancer. AJR Am J Roentgenol.

[5] Lawrence TS, Robertson JM, Anscher MS (1995). Hepatic toxicity resulting from cancer treatment. Int J Radiat Oncol Biol Phys.

[6] Liang SX, Zhu XD, Xu ZY (2006). Radiation-induced liver disease in three-dimensional conformal radiation therapy for primary liver carcinoma: the risk factors and hepatic radiation tolerance. Int J Radiat Oncol Biol Phys.

[7] Bujold A, Massey CA, Kim JJ (2013). Sequential phase I and II trials of stereotactic body radiotherapy for locally advanced hepatocellular carcinoma. J Clin Oncol.

[8] Jang WI, Kim MS, Bae SH (2013). High-dose stereotactic body radiotherapy correlates increased local control and overall survival in patients with inoperable hepatocellular carcinoma. Radiat Oncol.

[9] Kato H, Tsujii H, Miyamoto T (2004). Results of the first prospective study of carbon ion radiotherapy for hepatocellular carcinoma with liver cirrhosis. Int J Radiat Oncol Biol Phys.

[10] Mizumoto M, Okumura T, Hashimoto T (2012). Evaluation of liver function after proton beam therapy for hepatocellular carcinoma. Int J Radiat Oncol Biol Phys.

[11] Takeda A, Oku Y, Sanuki N (2012). Dose volume histogram analysis of focal liver reaction in follow-up multiphasic CT following stereotactic body radiotherapy for small hepatocellular carcinoma. Radiother Oncol.

[12] Kim TH, Kim DY, Park JW (2007). Dose–volumetric parameters predicting radiation-induced hepatic toxicity in unresectable hepatocellular carcinoma patients treated with three-dimensional conformal radiotherapy. Int J Radiat Oncol Biol Phys.

[13] Finkelstein JN, Johnston CJ, Baggs R (1994). Early alterations in extracellular matrix and transforming growth factor beta gene expression in mouse lung indicative of late radiation fibrosis. Int J Radiat Oncol Biol Phys.

[14] Katoh H, Ishikawa H, Hasegawa M (2010). Protective effect of urinary trypsin inhibitor on the development of radiation-induced lung fibrosis in mice. J Radiat Res.

[15] Hakenjos L, Bamberg M, Rodemann HP (2000). TGF-beta 1-mediated alterations of rat lung fibroblast differentiation resulting in the radiation-induced fibrotic phenotype. Int J Radiat Biol.

[16] Du SS, Qiang M, Zenq ZC (2010). Radiation-induced liver fibrosis is mitigated by gene therapy inhibiting transforming growth factor-β signaling in the rat. Int J Radiat Oncol Biol Phys.

[17] Seong J, Kim SH, Chung EJ (2000). Early alteration in TGF-β mRNA expression in irradiated rat liver. Int J Radiat Oncol Biol Phys.

[18] Kaplan E, Meier P (1958). Non-parametric estimation from incomplete observation. J Am Stat Assoc.

[19] Chung SI, Seong J, Park YN (2010). Identification of proteins indicating radiation-induced hepatic toxicity in cirrhotic rats. J Radiat Res.

[20] Chi CH, Liu IL, Lo BS (2005). Hepatocyte growth factor gene therapy prevents radiation-induced liver damage. World J Gastroenterol.

[21] Geraci JP, Mariano MS (1996). Radiation hepatology of the rat: association of the production of prostacyclin with radiation-induced hepatic fibrosis. Radiat Res.

[22] Ren ZG, Zhao JD, Gu K (2012). Hepatic proliferation after partial liver irradiation in Sprague-Dawley rats. Mol Biol Rep.

[23] Geraci JP, Mariano MS (1993). Radiation hepatology of the rat: parenchymal and nonparenchymal cell injury. Radiat Res.

[24] Mustoe TA, Pierce GF, Thomason A (1987). Accelerated healing of incisional wounds in rats induced by transforming growth factor-beta. Science.

[25] Misseri R, Rink RC, Meldrum DR (2004). Inflammatory mediators and growth factors in obstructive renal injury. J Surg Res.

[26] Nouchi T, Tanaka Y, Tsukada T (1991). Appearance of alpha-smooth-muscle-actin-positive cells in hepatic fibrosis. Liver.

[27] Chang XM, Chang Y, Jia A (2005). Effects of interferon-alpha on expression of hepatic stellate cell and transforming growth factor-beta1 and alpha-smooth muscle actin in rats with hepatic fibrosis. World J Gastroenterol.

[28] Saito S, Murase K (2012). Detection and early phase assessment of radiation-induced lung injury in mice using micro-CT. PLOS ONE.

[29] Verhaegen F, Granton P, Tryggestad E (2011). Small animal radiotherapy research platforms. Phys Med Biol.

